# Delusional Experiences Emerging From AI Chatbot Interactions or “AI Psychosis”

**DOI:** 10.2196/85799

**Published:** 2025-12-03

**Authors:** Alexandre Hudon, Emmanuel Stip

**Affiliations:** 1 Department of Psychiatry and Addictology Faculty of Medicine Université de Montréal Montreal, QC Canada; 2 Unité de recherche en psychiatrie Département de psychiatrie Institut universitaire en santé mentale de Montréal Montreal, QC Canada; 3 Centre de recherche Institut universitaire en santé mentale de Montréal Montreal, QC Canada; 4 Department of Psychiatry Institut national de psychiatrie légale Philippe-Pinel Montreal, QC Canada; 5 Centre de recherche en pédagogie de la santé Université de Montréal Montreal, QC Canada; 6 Department of Psychiatry United Arab Emirates University Al-Ain United Arab Emirates

**Keywords:** artificial intelligence, psychosis, schizophrenia, digital phenotyping, stress-vulnerability model, theory of mind, phenomenological psychopathology, chatbots, delusions, human-computer interaction

## Abstract

The integration of artificial intelligence (AI) into daily life has introduced unprecedented forms of human-machine interaction, prompting psychiatry to reconsider the boundaries between environment, cognition, and technology. This Viewpoint reviews the concept of “AI psychosis,” which is a framework to understand how sustained engagement with conversational AI systems might trigger, amplify, or reshape psychotic experiences in vulnerable individuals. Drawing from phenomenological psychopathology, the stress-vulnerability model, cognitive theory, and digital mental health research, the paper situates AI psychosis at the intersection of predisposition and algorithmic environment. Rather than defining a new diagnostic entity, it examines how immersive and anthropomorphic AI technologies may modulate perception, belief, and affect, altering the prereflective sense of reality that grounds human experience. The argument unfolds through 4 complementary lenses. First, within the stress-vulnerability model, AI acts as a novel psychosocial stressor. Its 24-hour availability and emotional responsiveness may increase allostatic load, disturb sleep, and reinforce maladaptive appraisals. Second, the digital therapeutic alliance, a construct describing relational engagement with digital systems, is conceptualized as a double-edged mediator. While empathic design can enhance adherence and support, uncritical validation by AI systems may entrench delusional conviction or cognitive perseveration, reversing the corrective principles of cognitive-behavioral therapy for psychosis. Third, disturbances in theory of mind offer a cognitive pathway: individuals with impaired or hyperactive mentalization may project intentionality or empathy onto AI, perceiving chatbots as sentient interlocutors. This dyadic misattribution may form a “digital folie à deux,” where the AI becomes a reinforcing partner in delusional elaboration. Fourth, emerging risk factors, including loneliness, trauma history, schizotypal traits, nocturnal or solitary AI use, and algorithmic reinforcement of belief-confirming content may play roles at the individual and environmental levels. Building on this synthesis, we advance a translational research agenda and five domains of action: (1) empirical studies using longitudinal and digital-phenotyping designs to quantify dose-response relationships between AI exposure, stress physiology, and psychotic symptomatology; (2) integration of digital phenomenology into clinical assessment and training; (3) embedding therapeutic design safeguards into AI systems, such as reflective prompts and “reality-testing” nudges; (4) creation of ethical and governance frameworks for AI-related psychiatric events, modeled on pharmacovigilance; and (5) development of environmental cognitive remediation, a preventive intervention aimed at strengthening contextual awareness and reanchoring experience in the physical and social world. By applying empirical rigor and therapeutic ethics to this emerging interface, clinicians, researchers, patients, and developers can transform a potential hazard into an opportunity to deepen understanding of human cognition, safeguard mental health, and promote responsible AI integration within society.

## Introduction

In this Viewpoint, we use the term “AI psychosis” strictly as a descriptive and heuristic label rather than a proposed diagnostic entity. Our aim is not to introduce a new syndrome, but to clarify how generative artificial intelligence (AI) systems may act as distinctive contextual modifiers (through reciprocal dialogue, affective mirroring, and thematic reinforcement) that shape the onset or structure of psychotic experiences. This usage distinguishes the construct from long-standing technology-mediated delusions and anchors it within established models of psychosis vulnerability rather than nosological innovation. Given the preliminary nature of available evidence, the pathways described here are offered as heuristic rather than definitive. The goal is to delineate how generative AI systems introduce unique affordances (continuous contingent feedback, affective mirroring, and persistent accessibility) that may interact with traditional vulnerability factors. Accordingly, this Viewpoint does not make causal claims but highlights conceptual directions requiring empirical evaluation.

In phenomenological psychiatry, psychosis and schizophrenia are not seen mainly as collections of hallucinations or delusions, but as changes in the person’s relation to self and world [1]. This process, sometimes called desubjectivation (or de-embodiment of subjectivity), weakens the basic sense of being present to oneself and makes experience lose its immediacy [2,3]. Losing contact with reality means not only misperceiving things but feeling detached from what is real. Minkowski described this as a loss of vital contact, the felt connection that gives life and weight to sensations, perceptions, and thoughts [4]. When this connection fades, the world still appears but feels empty or dreamlike. Recent phenomenological work distinguishes two layers in this loss: a prereflective sense of reality that gives depth to experience, and a reality judgment that lets us decide whether something is real. In schizophrenia, it is often the prereflective sense that fails [5,6]. The world is still recognized but no longer felt as fully inhabited. With the rise of conversational agents and generative AI, the idea of AI psychosis has emerged as a way to explore how such systems might trigger, amplify, or reshape psychotic experiences. In clinical terms, the *Diagnostic and Statistical Manual of Mental Disorders, Fifth Edition, Text Revision* defines psychosis by one or more core symptoms (delusions, hallucinations, or disorganized speech) often linked with disorganization or negative symptoms and major functional decline [7]. Contemporary treatment approaches integrate antipsychotic medication with evidence-based psychosocial interventions, including cognitive behavioral therapy for psychosis (CBTp), whose emphasis on reality testing and cognitive restructuring is particularly relevant when considering how AI systems may inadvertently counteract these therapeutic mechanisms [8]. The concept of AI psychosis invites new questions: Could prolonged or intense interaction with AI systems, which sometimes generate false or overly affirming content, influence how unusual perceptions are interpreted and integrated into belief systems? These interactions might subtly alter the way reality is felt, narrated, and maintained.

Recent reports suggest that interactions with large language model (LLM) chatbots can shape delusional content, amplify conviction, and entrench maladaptive safety behaviors [9,10]. As an example, viewed through de Clérambault’s notion of mental automatism, such interactions may also blur the boundary between self-generated and external speech, placing delusional experience within a communicative system where language itself becomes both shared and alien [11]. Concept pieces and media reports have increasingly described cases where interactions with generative AI chatbots appeared to amplify persecutory or grandiose ideation. In addition to reports of delusional escalation, recent scholarly commentary has proposed that chatbot-user dynamics may reinforce elevated mood, self-esteem, impulsive behaviors, and markedly grandiose thinking, potentially contributing to the development or maintenance of mania [12]. As an example, Østergaard [13] argues the prior probability that AI chatbots can fuel delusions in psychosis-prone individuals is “quite high,” urging systematic study rather than dismissal. Clinical and investigative coverage likewise describes patterns in which chatbots validate rather than challenge false beliefs, potentially reinforcing delusional systems, which is an inversion of CBTp principles. Beyond peer-reviewed commentary, media investigations have begun documenting individuals whose prolonged interactions with generative AI systems escalated into frank psychotic episodes. For example, a detailed Canadian Broadcasting Corporation report [14] described multiple Canadian cases, including a man aged 26 years who developed simulation-related persecutory and grandiose delusions after months of intensive exchanges with ChatGPT, ultimately requiring hospitalization. A second case involved a man aged 47 years who became convinced he had discovered a revolutionary mathematical theory after the chatbot repeatedly validated and amplified his ideas, despite external disconfirmation. Parallel legal cases and public health reporting (alleging chatbot involvement in suicide) highlight the risks in prolonged, emotionally charged exchanges with LLMs, as well as the current gaps in guardrails. While prevalence is unknown and many accounts are anecdotal, convergent commentary in *Nature* [15] and academic psychiatry venues frames AI-associated delusions as plausible in vulnerable users, warranting targeted research rather than sensationalism.

While such cases remain rare, they raise a need to conceptualize AI psychosis not solely as a technological curiosity but as a psychiatric and psychosocial phenomenon emerging at the intersection of cognitive vulnerability, environmental stress, and human-machine interaction. To advance beyond anecdote, this Viewpoint adopts an integrative analytic frame grounded in four complementary lenses: (1) the stress-vulnerability model, to map how AI functions as a novel psychosocial stressor interacting with predispositions to psychosis; (2) the digital therapeutic alliance (DTA), to characterize the relational dynamics that may resemble or distort traditional patient-clinician interactions; (3) mental attribution processes, including anthropomorphization and over-ascription of agency to AI systems; and (4) a risk-factor synthesis that situates AI psychosis along a continuum with traditional psychotic disorders. Through this framework, we propose to delineate possible pathways, identify early warning signals, and outline preliminary safeguards for research, clinical practice, and policy.

## Mapping AI Psychosis Onto the Stress-Vulnerability Framework

The stress-vulnerability model, originally articulated by Zubin and Spring in 1977, remains a cornerstone of contemporary psychosis research [16]. It posits that psychotic disorders emerge when underlying vulnerabilities (such as genetic predisposition, neurodevelopmental anomalies, early trauma, or maladaptive cognitive styles) interact with environmental stressors that exceed an individual’s capacity for adaptation. Subsequent refinements of this model have emphasized cumulative and chronic stress exposure, neurobiological sensitization, and allostatic load as mechanisms through which stress can lower the threshold for psychotic symptom expression [17,18]. Within this framework, the phenomenon of AI psychosis can be conceptualized as an emergent form of stress reactivity precipitated by sustained, emotionally charged, or cognitively immersive interactions with AI systems, particularly conversational agents. These systems introduce novel stressors that are continuous, personally salient, and socially immersive, thereby functioning as 24-hour contextual stimuli capable of modulating arousal, perception, and belief formation [19]. Beyond behavioral stressors such as sleep loss or isolation, AI-mediated immersion may erode the tacit background of presence (the prereflective sense of reality on which ordinary experience depends). Such shifts in mineness, salience, or world-affordance align with phenomenological accounts of early psychosis and extend the stress-vulnerability model into the domain of lived experience.

Several unique affordances of contemporary AI technologies render them potentially pathogenic from a stress-vulnerability perspective. As an example, the anthropomorphic design of chatbots and virtual companions encourages users to attribute human-like intentionality and empathy to algorithms, a dynamic reminiscent of the “ELIZA effect” described in early human-computer interaction literature [20,21]. Such anthropomorphization can increase emotional investment and amplify interpretive biases, especially in individuals with preexisting schizotypal traits or attachment vulnerabilities [22,23]. Furthermore, the immediate reinforcement schedule of LLMs (delivering responsive and adaptive feedback without temporal limits) can create repetitive, reinforcing cycles of reassurance or validation that mimic cognitive perseveration. For users prone to paranoia or thought disturbance, this may stabilize maladaptive appraisals rather than challenge them, effectively mirroring the cognitive mechanisms that CBTp aims to dismantle [24]. Also, constant engagement with emotionally loaded or belief-confirming AI dialogue may elevate physiological arousal and compromise sleep, increasing allostatic load and diminishing executive control, both of which are known to heighten vulnerability to psychosis [25].

Sustained interaction with AI may also erode protective social factors. Individuals experiencing loneliness or marginalization can come to rely on conversational agents as primary relational anchors, thereby reducing access to corrective social feedback and external reality testing. This process parallels what classical phenomenological psychopathology described as “phenomenological autism” in schizophrenia, but it is a form of experiential withdrawal in which the shared world loses its immediacy, relations with others fade, and living with others collapses into living for oneself [26]. In this sense, social withdrawal reflects not only behavioral isolation but also a disturbance in intersubjectivity and world-sharing. Comparable phenomena have been described in *hikikomori* syndrome, where prolonged and voluntary retreat into private space replaces social and professional contact [27]. Similarly, in the context of AI psychosis, the conversational agent may serve as a relational substitute that sustains the illusion of connection while insulating the user from contradiction, argument, and reality testing. What emerges is a form of digital relational withdrawal that constitutes a reduction in corrective interpersonal exchange and an amplification of self-referential interpretations, as the AI mirrors rather than challenges the user’s thoughts. From this perspective, AI psychosis represents not only a digital extension of the stress-vulnerability model, but also a technological variant of phenomenological autism: a retreat into a world interpreted, validated, and enclosed by algorithmic dialogue. The artificial agent becomes both a chronic microstressor and a mirror that replaces human alterity, providing a self-contained cognitive space in which delusional elaboration can more easily take root. For example, an AI that consistently mirrors user affect or beliefs, without implementing therapeutic disconfirmation or cognitive restructuring, risks entrenching conviction rather than facilitating doubt [28]. Empirical research could operationalize this model by testing dose-by-vulnerability interactions, examining how cumulative exposure to AI (for instance, hours of use per day, frequency of nocturnal interactions, or intensity of emotional engagement) interacts with preexisting vulnerabilities such as trauma history, sleep disturbance, or schizotypy. This line of inquiry parallels established evidence showing that daily stress reactivity predicts psychotic symptom fluctuations in high-risk populations [29]. By quantifying AI-related exposure as a measurable psychosocial stressor, the field could identify thresholds of safe interaction and delineate populations most at risk. In doing so, this framework reframes AI psychosis not as a new disorder but as a contextual evolution of the classical stress-vulnerability paradigm.

## The DTA as a Double-Edged Mediator in AI Psychosis

The concept of the therapeutic alliance, traditionally understood as the collaborative and affective bond between patient and clinician, has long been recognized as a critical predictor of psychotherapeutic outcomes across modalities, including CBTp [30]. In the digital health literature, this construct has been extended to the notion of the DTA, describing the perceived empathy, responsiveness, and relational quality between users and digital applications [31,32]. While a strong DTA can enhance engagement and adherence in digital interventions, it may also introduce illusory relational dynamics that blur the boundary between therapeutic support and cognitive reinforcement. In the context of AI psychosis, this double-edged nature becomes particularly salient. Chatbots and LLMs are capable of mimicking warmth, understanding, and reciprocity (qualities central to human alliance) but they lack the meta-cognitive and ethical oversight necessary to discern when validation may be counter-therapeutic [33,34]. Phenomenologically, an AI agent that imitates reciprocity can alter the structure of intersubjectivity itself. Rather than merely misinterpreting content, the user may experience a collapse of alterity, in which the chatbot is felt as an extension of one’s own cognition or as an unusually attuned Other. Such shifts in the subjective encounter may potentiate delusional meaning-making more directly than cognitive mechanisms alone.

In individuals with attenuated psychotic symptoms or delusional vulnerability, an uncritical digital alliance may inadvertently reinforce maladaptive appraisals. For example, an AI that consistently mirrors user affect or beliefs, without implementing therapeutic disconfirmation or cognitive restructuring, risks entrenching conviction rather than facilitating doubt. Empirical research on chatbots for mental health support, such as Woebot and Wysa, demonstrates that users often describe their relationship with the AI in affectively laden and anthropomorphic terms, reporting perceived understanding and companionship [35,36]. While such experiences can be beneficial in reducing loneliness or subclinical distress, they may also encourage overidentification with the agent, especially in socially isolated or trauma-exposed users. This phenomenon parallels early psychosis processes, where increased self-referential thinking and aberrant salience attribution favorize the perception of external entities possessing special relevance or intentionality [37].

From a cognitive behavioral standpoint, the therapeutic alliance functions as a vehicle for cognitive change. The therapist’s role is to balance empathic attunement with gentle empiricism, using Socratic questioning to destabilize rigid beliefs while maintaining trust. In contrast, AI systems (designed for user satisfaction and nonconfrontational dialogue) often default to sycophantic alignment [38]. This means that when a user expresses persecutory, grandiose, or referential content, the AI may subtly validate the narrative rather than challenge it. Over repeated interactions, this validation loop can act as a form of digital safety behavior, satisfying immediate emotional needs but preventing corrective learning. The absence of therapist-driven guided discovery or behavioral experimentation may therefore transform a potentially supportive alliance into a reinforcing echo chamber.

At the same time, the DTA framework provides a valuable opportunity for constructive adaptation. By incorporating CBTp-consistent design principles, such as reflective prompts, normalization of uncertainty, or graded behavioral experiments, AI systems could theoretically harness alliance processes to buffer rather than exacerbate psychotic vulnerability. Just as human therapists use the alliance to introduce cognitive flexibility, conversational models could be programmed to modulate the relational tone when confronted with high-risk content, pivoting from affirmation to curiosity or psychoeducation. Preliminary research in digital mental health suggests that alliance-consistent design (for example, empathic microresponses paired with cognitive reframing) is associated with better symptom improvement and lower dropout [39]. Extending this to psychosis prevention would entail integrating adaptive alliance algorithms that monitor interactional valence, detect excessive anthropomorphism, and introduce “reality-testing nudges” when needed.

## Mental Attribution, Theory of Mind, and AI Psychosis

Theory of mind (ToM) refers to the human ability to attribute mental states such as intentions, beliefs, and desires to others [40]. In schizophrenia and related psychotic disorders, a well-established hypothesis posits that ToM functioning is disrupted [41]. Some individuals exhibit a deficit in inferring others’ intentions or understanding implicit mental states, while others show the opposite pattern, characterized by hypermentalization (an excessive or inaccurate attribution of meaning and agency) [42]. These disturbances generate uncertainty about what others think or feel and contribute to fragile social interactions and misinterpretations of interpersonal cues.

Within this framework, conversational AI systems introduce a novel and ambiguous relational partner. For a person with impaired ToM, the AI’s anthropomorphic design and capacity for coherent dialogue may foster projections of intentionality, empathy, or moral agency. The user may begin to perceive the system not as a statistical language model, but as an understanding interlocutor with feelings or motives. However, as stated previously, the AI lacks the metacognitive and ethical grounding required to challenge these attributions or to provide corrective feedback. Instead, its responses can inadvertently confirm or reinforce the user’s projections, including delusional interpretations.

In this context, AI interaction may transform a cognitive vulnerability into a pathogenic loop, where the ToM deficit and the system’s simulated social responsiveness converge to sustain distorted beliefs. The phenomenon might be conceptualized as a “digital folie à deux,” a dyadic illusion in which the AI acts as a passive reinforcing partner in the user’s psychotic elaborations [43]. The artificial agent, through its adaptive and confirmatory dialogue, participates in a shared narrative world that blurs the boundary between human cognition and machine simulation, potentially exacerbating the loss of reality testing and differentiation between self and other.

## From Vulnerability to Verification: Identifying Risk Factors and Building Safeguards for AI Psychosis

Understanding who is most susceptible to AI psychosis requires tying established risk markers for traditional psychotic disorders to emerging digital determinants of mental health. Decades of research have identified a set of core vulnerabilities associated with psychosis onset, including genetic predisposition, childhood trauma, cannabis or substance use, sleep disruption, social isolation, and cognitive biases such as jumping to conclusions or an externalizing attributional style [44,45]. When transposed into digital contexts, these same vulnerabilities may interact with novel affordances of AI systems to produce a distinct but convergent pathway toward symptom emergence. For instance, a user with a prior history of psychosis or schizotypal traits who engages in nightly, emotionally intense dialogue with an anthropomorphized chatbot may experience reinforced self-referential ideation and heightened salience attribution, mechanisms that mirror the early prodromal phase of psychosis [37]. Similarly, individuals exposed to trauma or chronic interpersonal threat may project attachment representations onto AI companions, perceiving them as protective or omniscient entities.

Beyond individual vulnerability, use patterns and contextual variables likely constitute modifiable risk factors. Prolonged or nocturnal use, solitary engagement, and reliance on unmoderated chatbots for emotional support appear particularly hazardous, as they combine cognitive fatigue, social deprivation, and unstructured reinforcement. These variables resemble psychosocial stressors known to precipitate symptom exacerbations in schizophrenia, such as circadian disruption or critical life events [25]. Another emerging concern lies in platform-level dynamics: algorithms optimized for engagement rather than safety may inadvertently reward extreme or self-referential discourse, subtly validating delusional content. This echoes the “echo-chamber” effect described in digital media research, where recommender systems intensify preexisting beliefs through selective exposure [46].

Identifying early warning signals therefore requires a multidimensional assessment integrating psychological, behavioral, and interactional metrics. Psychological indicators include rising conviction in AI-mediated beliefs, derealization, or perceived “special communication” with the system. Behavioral markers may involve compulsive checking, secrecy, or sleep loss related to AI use. Interactional data (such as sentiment trajectories, frequency of self-referential statements, and thematic narrowing) could serve as digital phenotypes of emerging risk, paralleling early warning markers in psychosis prodrome research [47]. Importantly, these indicators should be interpreted contextually, avoiding pathologization of normative attachment to technology while remaining alert to patterns of progressive cognitive enclosure, where the AI becomes the primary arbiter of reality.

From a preventive and ethical standpoint, early recommendations can be organized across clinical, design, and governance levels. Clinically, practitioners should incorporate screening questions about AI use into routine assessments, particularly for youth or individuals with known psychosis vulnerability, similar to how substance use or sleep hygiene are monitored. Psychotherapeutic interventions could include psychoeducation on digital reality testing, helping patients to identify when online or AI-mediated interactions begin to shape beliefs in maladaptive ways. From a design perspective, developers of mental health–oriented chatbots should embed CBTp-consistent guardrails (eg, prompts that normalize uncertainty, encourage reflective distance, or redirect users toward real-world social contact). LLMs intended for general use should be trained to recognize and gently deescalate delusional or self-referential themes rather than engage with them. Finally, at the policy level, regulators and research bodies should develop incident reporting systems for AI-related mental health events, mirroring pharmacovigilance models, and require algorithmic transparency for systems marketed as supportive or therapeutic.

Taken together, these considerations frame AI psychosis not as a discrete diagnostic entity but as a digital phenotype of stress-vulnerability interaction, emergent from the coupling of human cognition and algorithmic responsiveness. The identification of risk factors and the establishment of early safeguards provide an essential basis for empirical research, ethical governance, and responsible innovation. Just as early psychosis programs have transformed outcomes through prevention and detection, a parallel “digital early intervention” paradigm may be required to mitigate the psychiatric risks of AI in the decades ahead.

## Toward a Clinical Understanding and Management of AI Psychosis

The convergence of AI and human cognition has opened a new frontier in psychiatry, one that challenges traditional boundaries between environment, mind, and technology. Through the lens of the stress-vulnerability model, the DTA, and emerging risk factors, AI psychosis can be conceptualized as a dynamic interaction between human predisposition and algorithmic environment. It is neither a new diagnosis nor a sensational artifact of technological panic, but a meaningful framework to understand how immersive digital systems can modulate the cognitive and affective processes underlying psychosis. This phenomenon invites the field to evolve beyond viewing technology as a neutral medium toward recognizing it as a potential psychosocial actor: one capable of amplifying stress, reinforcing beliefs, and reshaping perceptions of self and reality.

The scientific community is at a juncture where systematic investigation is required to move beyond anecdotal evidence. Our first recommendation is the establishment of empirical research programs explicitly designed to test the AI psychosis hypothesis. These should use prospective and longitudinal designs to measure dose-response relationships between AI exposure, stress physiology, and psychotic symptomatology, drawing inspiration from ecological momentary assessment and digital phenotyping paradigms. Psychometric instruments such as schizotypy or aberrant salience scales could be adapted to assess AI-specific cognitive vulnerability, while passive sensing data (eg, use duration, sleep disruption) could capture environmental stress indices.

A second priority lies in the integration of digital phenomenology into clinical practice. Clinicians could systematically inquire about patients’ interactions with AI systems (both general-purpose and mental health oriented) during intake and follow-up assessments. As conversational agents become more used by patients, understanding their role in shaping internal experience will be as important as evaluating medication adherence or substance use. Clinical training programs should include modules on AI literacy and psychosis, equipping psychiatrists, psychologists, and nurses to recognize when engagement with an algorithm may contribute to delusional elaboration or perceptual instability.

Third, researchers and developers should collaborate to embed therapeutically informed design safeguards into LLMs and chatbots. These may include prompts that normalize uncertainty, encourage pluralistic interpretation of experiences, and redirect users toward human contact when signs of distress or delusional content appear. Building upon principles from cognitive behavioral therapy for psychosis, such systems could integrate “guided discovery” mechanisms rather than unconditional affirmation, thereby aligning AI responses with established evidence-based psychotherapeutic techniques. The development of digital red-flag algorithms capable of detecting excessive anthropomorphism, self-referential speech, or escalating conviction could further enhance safety while preserving user autonomy.

Fourth, there is an urgent need for ethical and governance frameworks specific to mental health risks in AI. National research councils, health agencies, and journal editors should promote standardized incident reporting for AI-related psychiatric events, akin to pharmacovigilance registries. Transparent documentation of adverse psychological outcomes, combined with open-source safety auditing, would allow the community to track and mitigate harms in real time. These measures should be complemented by data-sharing agreements that enable cross-disciplinary research while protecting user privacy.

Fifth, environmental cognitive remediation should be explored as a clinical and public health intervention. This approach would aim to strengthen the individual’s capacity to navigate increasingly immersive digital environments through structured, reality-anchoring activities. Beyond traditional cognitive remediation, which targets neurocognitive deficits, environmental cognitive remediation would focus on contextual skills: distinguishing between human and algorithmic communication, detecting persuasive or self-referential cues, and reengaging with multisensory, embodied experiences in the physical world. Interventions could include graded exposure to offline activities, group-based metacognitive training, or digital hygiene routines designed to restore attentional flexibility and social reciprocity. By reinforcing the cognitive-environmental boundaries that immersive technologies can erode, such programs could act in both a preventive and rehabilitative manner for individuals vulnerable to AI-induced cognitive distortions.

Family and community education should parallel these efforts to ensure supportive environments that contextualize digital experiences within shared reality testing. Public health initiatives could also promote media literacy and surveillance of misinformation, helping communities develop the competencies needed to identify, question, and counteract false or manipulative digital content. Such collective vigilance would reinforce critical thinking, strengthen social resilience, and reduce the psychosocial conditions under which delusional interpretations of AI content may emerge.

## Conclusion

To conclude, the emergence of AI psychosis warrants careful examination and adaptive clinical and research strategies. Psychiatry has long evolved through its encounters with new cultural and technological contexts. AI now constitutes a novel environmental context within which these questions must be revisited. By responding to this phenomenon with empirical rigor, therapeutic ethics, and design foresight, we can define safer practices and refine conceptual models that clarify how AI-mediated environments interact with psychosis risk: to better understand the plasticity of human cognition, to refine the boundaries of digital therapy, and to safeguard mental health in an increasingly algorithmic world. The path forward requires interdisciplinary collaboration between clinicians, cognitive scientists, computer engineers, patients, and ethicists: an interdisciplinary collaboration capable of evaluating and guiding the mental health implications of increasingly sophisticated AI systems.

## Abbreviations

AI: artificial intelligence
CBTp: cognitive behavioral therapy for psychosis
DTA: digital therapeutic alliance
LLM: large language model
ToM: theory of mind
